# Smoking cessation after referral from hospital to community stop smoking services: an observational study

**DOI:** 10.1136/bmjph-2024-001659

**Published:** 2025-11-06

**Authors:** Rowan Cherodian, Matthew Franklin, Susan Baxter, James Chilcott, Duncan Gillespie

**Affiliations:** Sheffield Centre for Health and Related Research (SCHARR), School of Medicine and Population Health, The University of Sheffield, Sheffield, UK

**Keywords:** statistics and numerical data, Public Health, Community Health

## Abstract

**Introduction:**

In England, acute National Health Service (NHS) hospitals routinely ask patients about smoking status on admission, offering in-hospital treatment for tobacco dependence and support for quitting postdischarge. Referring patients to community stop smoking services (CSSS), which offer behavioural and pharmacological support postdischarge, is a key strategy for this continued support. This study investigated the patient flows from hospital to CSSS and the subsequent quitting outcomes.

**Methods:**

This study was part of an evaluation of a hospital tobacco dependence treatment service in South Yorkshire, England. The primary data source was electronic record data from one CSSS that received hospital referrals. Data were from July 2021 to March 2023, covering the initial phase of hospital service implementation. We described patient flows from hospital referral through to the 4-week self-reported quitting outcomes recorded by the CSSS. Generalised linear models explored associations between 4-week abstinence and patient characteristics including demographics, socioeconomic status, nicotine dependence and health factors.

**Results:**

Of 3223 hospital referrals, 72.0% (2322) could be contacted by the CSSS, 52.5% (1692) then registered, 41.4% (1333) made a CSSS-supported quit attempt and 25.3% (815) self-reported abstinence from smoking 4 weeks later. The analysis highlighted lower quitting success for people receiving free NHS prescriptions—an indicator of health and/or socioeconomic vulnerability (OR 0.54, 95% CI 0.32 to 0.90) and with high nicotine dependence (OR 0.57, 95% CI 0.37 to 0.87). Higher quitting success was found for people who reported having cancer (OR 2.26, 95% CI 1.18 to 4.32), but otherwise, there were no significant influences of the health factors investigated.

**Conclusions:**

The substantial drop-out between hospital referral to CSSS and receiving their support for quit attempts is a key area for hospital and CSSS service improvement. The strong quitting success among people with cancer underscores the potential benefits of improved care transfer for vulnerable patient groups.

**WHAT IS ALREADY KNOWN ON THIS TOPIC**

- Hospital-based support helps patients try to quit smoking; however, their long-term success may depend on whether they use high-quality counselling and stop smoking pharmacotherapy after leaving the hospital.

**WHAT THIS STUDY ADDS**

- Our study found that while many patients referred for this support can be contacted, fewer actually sign up, and only about a quarter remain smoke-free after 4 weeks.
- People eligible for free prescriptions (indicating vulnerability) or with high nicotine dependence were less likely to quit successfully.
- However, those with cancer were more likely to succeed, but other health factors were not found to have a significant influence.

**HOW THIS STUDY MIGHT AFFECT RESEARCH, PRACTICE OR POLICY**

- The findings highlight the need to reduce patient drop-out between hospital referral and starting high-quality support to stop smoking in the community.

## Introduction

Tobacco smoking remains a leading cause of morbidity and mortality worldwide.^1^ In the UK, smoking prevalence has been declining over decades due to public investment in population-level tobacco control measures and individually tailored smoking cessation support services.^2–4^ However, further investment is still needed, with particular attention to new interventions that reach people in vulnerable population groups who might not otherwise engage with support to stop smoking.^5 6^

The 2019 Long Term Plan for the National Health Service (NHS) in England set the goal of introducing NHS-funded tobacco dependence treatment services for all patients admitted to hospital who currently smoke.^7^ Since then, many hospital tobacco dependence treatment services have been introduced across England, with similar services in Scotland, Wales and Northern Ireland. A key part of the rationale behind these services is that a hospital contact for someone who smokes, particularly for a smoking-related disease, can be a moment in which people are more open to considering quitting smoking.^8 9^ Furthermore, the opt-out nature of the service—in which tobacco dependence treatment is automatically offered to all—is likely to increase equity in access to smoking cessation support.

The intervention relies on a high percentage of patients admitted to hospital being asked if they smoke. Patients identified as currently smoking are then given initially brief advice on the available support to stop smoking and the hospital’s smoke-free policy, with an automatic referral to an in-hospital tobacco dependence treatment advisor. Patients who stay in hospital for more than a day are then supposed to receive a specialist assessment from a tobacco dependence treatment advisor, who takes time to understand their situation, offer support to remain smoke-free during the hospital stay and incorporate treatment of tobacco dependency into their personal care plans.^10 11^ As part of this support, patients who commit to making a quit attempt on discharge are asked if they would like to be referred for continued support to stop smoking in the community. There are a number of options for this post-discharge support—including continued engagement with the hospital tobacco team by telephone or referral to local authority funded community stop smoking services (CSSS). Evidence suggests that connecting patients to effective support to remain smoke-free after discharge is vital for the success of hospital tobacco dependence treatment services.^12^ For example, in the Ottawa model for smoking cessation—which is used as the exemplar for the English services—patients received eight phone calls from the hospital tobacco team over a period of 6 months after discharge.^13 14^ The CURE pilot of these services in Wythenshawe hospital in Manchester included 12 weeks of follow-up support.^15^ However, the national roll-out of hospital tobacco dependence treatment services presents a challenge of how to effectively connect patients to postdischarge support,^16–18^ especially considering the diverse and uneven provision of community-based support to stop smoking around the country.^10^

Since 2013, CSSS have been funded by local governments in England and are generally commissioned to support anybody in the local area who is seeking quit support.^19 20^ In 2023, 63% of local governments in England commissioned CSSSs,^21^ and there have been recent initiatives to increase the national coverage of CSSS provision and to improve access to these services by priority population groups.^22^ Referral of patients to CSSS from hospital tobacco dependence treatment services could potentially represent a large percentage of CSSS activity, but could also bring challenges of how to engage and support people who may have long-term health conditions to stop smoking.^18 19^

This study is part of an evaluation of the QUIT hospital-based tobacco dependence treatment programme in South Yorkshire, England (https://sybics-quit.co.uk; see section 1 of the online supplemental information).^23^ The scope of this study extends beyond the hospital-based service to investigate the success in quitting smoking of patients who were referred (or self-referred) to CSSS following their contact with hospital.^23^ The study had three objectives: (1) to describe the flows of patients from the hospital service to the CSSS, including estimating the impact of these referrals on CSSS activity; (2) to quantify the quitting success rates of hospital-referred patients who receive CSSS support, comparing this to the general cohort of people making a CSSS-supported quit attempt and (3) to statistically investigate the factors that influenced quitting success among hospital-referred individuals. For (3), we used regression analyses to identify factors associated with quitting success, investigating the influence of patient health and sociodemographic characteristics and the nature of their contact with the hospital-based service.

10.1136/bmjph-2024-001659.supp1 Supplementary data

## Materials and methods

### Patient and public involvement

Analysis and interpretation were informed by the wider QUIT evaluation, which included patient surveys, interviews/workshops with QUIT and CSSS staff, and regular engagement with QUIT service managers.

### The hospital service and its link to CSSS

The QUIT tobacco dependence treatment service—involving four acute hospitals, three specialist mental health hospitals and one children’s hospital—began in May 2021.^23^ The service does not cover maternity wards, which have a separate stop smoking service, but does offer support to NHS staff who smoke. This study investigates patient flows and quitting outcomes for patients referred from an acute hospital to the NHS Yorkshire Smokefree CSSS (https://yorkshiresmokefree.nhs.uk/) over a 21-month period: July 2021 to March 2023. The three hospitals referring to this CSSS were Barnsley Hospital NHS Foundation Trust, Doncaster and Bassetlaw Hospitals NHS Foundation Trust and Sheffield Teaching Hospitals NHS Foundation Trust. A fourth hospital—The Rotherham Hospital NHS Foundation Trust—referred to a different CSSS, but data from this CSSS were not available. Both inpatients and outpatients could be referred to CSSS, or given information to self-refer. Patients could receive only Very Brief Advice or a specialist assessment involving motivational interviewing by a hospital tobacco dependence treatment advisor.^10^ For recently discharged inpatients, the hospital team gives a follow-up call to patients who had an assessment. In addition, if a patient is discharged before seeing the hospital tobacco team, they can also be called and offered a specialist assessment remotely.

### Data

This study used four data sources. First, individual-level data (July 2021 to March 2023) on quitting outcomes with the CSSS. Second, summary data (July 2021 to March 2023) on patient flows from hospital to the CSSS through to making a CSSS-supported quit attempt (online supplemental table S1). Third, summary data (November 2022 to March 2023) from the three referring hospitals, describing patient flows through the inpatient tobacco dependence treatment pathway. Fourth, publicly available local and national CSSS reporting data (April 2022 to March 2023), which we used for comparison (online supplemental tables S2 and S3).^24^ In these data, the Yorkshire and the Humber region (that includes the CSSS in this study) tends to have the highest quit rates nationally.^24^

### Outcome measure: postdischarge CSSS-supported 4-week quit

A ‘4-week quit’ is defined according to the nationally adopted standard, as an individual not having smoked at all in the last 2 weeks when asked at 4 weeks (28 days) from their ‘quit date’, marking the start of a quit attempt for the purposes of outcome monitoring.^25^ A person is counted as having achieved a self-reported 4-week quit if they are assessed (face-to-face or by telephone) and state that they have not smoked according to this standard (note that self-reporting tends to over-estimate rates of quitting).^26^ During the study period, carbon monoxide (CO) monitoring of quits was not required due to COVID-19-related adaptations to the service. CO monitoring might also not be appropriate for people feeling unwell. Quits were therefore counted if either self-reported or CO validated.

For inpatients who had a specialist assessment by a hospital-based tobacco dependence treatment advisor, the hospital discharge date is initially set as the patient’s quit date, marking the start of the quit attempt. If the patient was transferred to the CSSS, and they had smoked since discharge, then a new quit date is agreed with the CSSS. If a patient relapsed back to smoking while under CSSS care, then they could reset their quit date. We used data on quitting outcomes at 4 weeks after setting the latest quit date. Individuals are recorded as either ‘quit’, ‘lost to follow-up’ (LTF) or ‘not quit’. For our primary analysis, all ‘LTF’ were set as ‘not quit’, a conservative assumption. Rather than imputing missing quitting outcome data, which can be prone to bias,^27^ we instead repeated our analysis excluding people recorded as LTF.

### Explanatory variables for regression analyses

Explanatory variables were determined through a systematic process,^23^ including a literature review^28^ and cross-referencing with available data fields. The resulting variables are described below with further information in online supplemental tables S4–S6.

#### Demographic and socioeconomic variables

We investigated the effects of age (18–34, 35–44, 45–59, 60+), sex and occupation. Information on the Index of Multiple Deprivation—a small-area geographic indicator of socioeconomic conditions—was not available. Instead, we used another composite indicator—exemption from NHS prescription payments. In England, someone is eligible for free NHS prescriptions if they meet certain criteria, including being aged 60+, pregnant or a recent mother, having a specified disability or medical condition, or receiving social welfare benefits.^29^

#### Strength of nicotine dependence

The Fagerström score for nicotine dependence was dichotomised into a binary variable representing low/medium (0–5) and high (6+) nicotine dependence.^30^

#### CSSS support

Support was characterised by the number of support sessions attended and, for pharmacological support, an index that we derived to represent the intensity of nicotine replacement therapy (NRT) supplied.^23^ During the study period, varenicline was not available,^31^ and e-cigarettes, although widely used in England, were not at the time routinely supplied by the CSSS. In their sessions, people could be recorded as receiving up to two types of NRT, such as patches, sprays and lozenges in different quantities, with most receiving a combination of types over the course of their sessions. To simplify the NRT provided for analysis purposes, an intensity index of the NRT supplied was calculated by dividing the total number of times NRT was given by the number of sessions attended—higher values indicating more intensive pharmacotherapy. We dichotomised this index into people who on average received ≤1 NRT items per session vs >1 item.

#### Type of contact with the hospital-based service

Patient contact with the hospital-based service was described by a single variable with four categories:

1. Specialist assessment conducted in person during the inpatient stay.
2. Specialist assessment or Very Brief Advice over the phone postdischarge.
3. Specialist assessment or Very Brief Advice given as an outpatient.
4. Unknown.

#### Health variables

When registering with the CSSS, someone could report having (or not) any of 16 medical conditions (with no additional details, for example, subcondition type, treatment type, time of diagnosis). These conditions were grouped to produce: (1) five binary variables for condition category: chronic respiratory, cardiovascular disease, diabetes, mental health and cancer; (2) a comorbidities variable with three categories: 0, 1–2, 3+ conditions.

### Data analysis

The data sample comprised 1677 individuals registered with the CSSS. From this sample, 1641 individuals (98%) were retained, excluding under 18s (n=2), parents/carers of children admitted to hospital (n=24) and NHS staff (n=10). We further restricted the sample to 1332 individuals (81%) who had a CSSS support session and set a start date for their quit attempt. We also excluded 6 individuals with missing data on occupation, leaving an analysis sample of 1326.

Data analysis followed the preregistered plan,^32^ with refinements for data limitations. Data missingness with respect to quitting outcomes was assessed using two-way t-tests, with the results informing the approach to missingness.^33^ Analysis of 4-week quitting success used generalised linear models with logit link functions. Five model structures were investigated, sequentially adding explanatory variables:

- (Model 1) individual demographic and socioeconomic characteristics.
- (Model 2) Model 1 with the Fagerström score for nicotine dependence.
- (Model 3) Model 2 with CSSS support (support sessions and pharmacotherapy).
- (Model 4) Model 3 with hospital contact type (inpatient vs postdischarge vs outpatient).
- (Model 5) Model 4 with health comorbidities.

Models were compared using Akaike’s information criterion and likelihood ratio tests of the difference in residual deviance. Statistical effect sizes are reported in terms of adjusted ORs. The threshold for statistical significance was set at p=0.05 (two-way), producing 95% CIs, which were adjusted for the multiple imputation of missing data using Rubin’s rule.^34^

## Results

### Patient flows through hospital and CSSS

Of the 3223 patients referred to the CSSS from hospital, 2322 (72.0%) could subsequently be contacted, 1692 (52.5%) went on to register with the CSSS, 1333 (41.4%) had a CSSS recorded quit date and 815 (25.3%) were recorded to still be smoke-free 4 weeks later (table 1). For the first 10 months of implementation of the hospital tobacco dependence treatment service, there was a steady rise in postdischarge referrals to CSSS (online supplemental figure S1). After this initial period, from April 2022, the monthly statistics were relatively stable, with the hospital service accounting for an average of 26% of all CSSS referrals (191 referrals/month), translating to 19% of quit dates recorded (81 quit dates/month) and 17% of 4-week quits (49 quits/month).

**Table 1:** CSSS statistics on patients referred from hospital

	Count	Stepwise percentage	Percentage of referrals
Number of referrals from the (QUIT) hospital service to CSSS	3223		
Contactable by the CSSS	2322	72.0	72.0
Registered with the CSSS	1692	72.9	52.5
Set a quit date with the CSSS, marking the start of a quit attempt	1333	78.8	41.4
Recorded by the CSSS to be smoke-free 4 weeks after beginning the quit attempt	815	61.1	25.3

These are summary statistics provided by the NHS Yorkshire Smokefree CSSS and are separate to the analysis data sample. The data in this table correspond to the period July 2021 to March 2023.

CSSS, community stop smoking service; NHS, National Health Service.

Put in the context of patient flows through the inpatient tobacco dependence treatment pathway (table 2), on average across the three hospitals, for every 100 000 inpatient admissions, 61 793 people were asked if they smoke on admission, 9683 were identified as currently smoking, of whom 4112 had a specialist assessment by a hospital tobacco dependence treatment advisor. In these assessments, 1248 people consented to be referred to a local CSSS after discharge. These referrals then led to 517 CSSS-supported quit attempts and 316 4-week quits (table 1). This equates to a CSSS-supported quit rate of 3.3% of inpatients identified as smoking (range 1.3% to 4.7% across the three hospitals), 7.7% of inpatients who had a specialist assessment (range 6.2% to 9.8%) and 25.3% of inpatients referred to a CSSS (no range due to only one CSSS).

**Table 2:** Main hospital inpatient pathway for the three acute hospitals that referred patients to the CSSS in this study

	Barnsley Hospital NHS Foundation Trust		Doncaster and Bassetlaw Hospitals NHS Foundation Trust		Sheffield Teaching Hospitals NHS Foundation Trust		Total	
	Number	%	Number	%	Number	%	Number	%
Number of admissions	8974		11 084		25 546		45 604	
Number asked if they smoke*	6479	72.2	10 609	95.7	11 092	43.4	28 180	61.8
Number identified as currently smoking†	868	13.4	1638	15.4	1910	17.2	4416	15.7
Number of hospital-based tobacco treatment advisor specialist assessments‡	337	38.8	339	20.7	1204	63.0	1880	42.6
Number of specialist assessments for locally resident patients§	329	97.6	222	65.5	1159	96.3	1710	91.0
Number referred to CSSS§	130	39.5	83	37.4	356	30.7	569	33.3

Data from November 2022 to March 2023. Data are limited to inpatients aged 16 and over with a length of stay ≥1 day. These figures are an excerpt from the monthly monitoring data collated by the South Yorkshire Integrated Care Board.

*Patient smoking status electronically recorded in the nursing records within 24 hours of admission.

†This refers to people identified as currently smoking by either nursing staff or a hospital-based tobacco dependence treatment advisor at any point during their admission.

‡This could be a specialist assessment either in person while an inpatient or a post-discharge phone call.

§This refers to people who were registered with a general practitioner within the South Yorkshire and Bassetlaw Clinical Commissioning Group (CCG) area. While CCGs as an organisational entity have since been replaced by Integrated Care Boards, whether someone was registered in the local CCG area is a useful indicator that they are likely to attend the local CSSS, as opposed to a CSSS in another part of the country.

CSSS, community stop smoking service; NHS, National Health Service.

### Descriptive statistics and comparison to the local and national samples

Table 3 describes the analysis sample with respect to quitting success and the explanatory variables investigated in the statistical analysis.

**Table 3:** Description of the analysis sample

	Number of observations	Percentage
Total	1326	
Quitting outcome		
4-week quit achieved*	813	61.3
Demographic and socioeconomic variables		
Sex (male, female)		
Male	691	52.1
Age		
18–34	112	8.5
35–44	190	14.3
45–59	504	38.0
60+	520	39.2
Occupation		
Routine and manual occupations	411	31.0
Retired	321	24.2
Sick/disabled and unable to work	297	22.3
Never worked or unemployed for over 1 year	196	14.8
Other	102	7.7
Eligible for free NHS prescriptions	1076	81.1
Strength of nicotine dependence (Fagerström score) †		
0–5	738	55.7
6+	588	44.3
Community stop smoking service support		
Number of support sessions attended (whole sample average)		6.3 (range: 1 to 26)
Nicotine replacement therapy items per session (≤1 item, >1 item)		
>1 item	412	31.0
Type of contact with the hospital-based service		
Specialist assessment in-person while an inpatient	584	44.0
Postdischarge specialist assessment or Very Brief Advice	66	5.0
Outpatient specialist assessment or Very Brief Advice	173	13.1
Unknown	503	37.9
Health variables		
Specific health conditions (% present vs not present)		
Chronic respiratory condition	498	37.6
Mental ill health	349	26.3
Cardiovascular disease	334	25.2
Diabetes	165	12.4
Cancer	123	9.3
Number of comorbid health conditions		
0	373	28.1
1–2	616	46.5
3+	337	25.4

See online supplemental tables S2 and S3 for comparisons to the local and national profiles of CSSS support quit attempts and subsequent quitting success.

*10.0% of the sample were lost to follow-up and so had unknown quitting outcomes.

†Missing values imputed; statistics generated from the average of 19 imputed data sets, each with N=1326.

CSSS, community stop smoking service.

Compared with the national data on people attempting to quit smoking with CSSS support, our data sample of people referred from hospital had a higher frequency of people who were retired, sick or disabled and unable to work, or eligible for free NHS prescriptions. Of the hospital-referred people who made a CSSS-supported quit attempt, 61.3% achieved a 4-week quit (n=813/1326). Compared with the local average CSSS supported quit rate of 69.6% (3540/5,083), the odds of quitting in people referred from hospital were 30.9% lower (χ^2^=33.1, df=1, p<0.01). However, quitting success in our hospital-referred data sample was similar to national averages.

### Missing data assessment

Missing data were mainly associated with the Fagerström score which had 252 missing values (19% of the analysis sample). This missingness was assessed to be ‘missing at random’ with respect to quitting outcomes (non-missing 61.9%, missing 58.7%, *t*-statistic=−1.026, df=251, p=0.306). Missing values for the Fagerström score were therefore imputed using multivariate imputation, based on information from all other analysis dataset variables.^33^ The result was nineteen imputed datasets (same as the percentage missing), accounting for the statistical uncertainty in imputation.

### Statistical analyses

**Figure 1:**
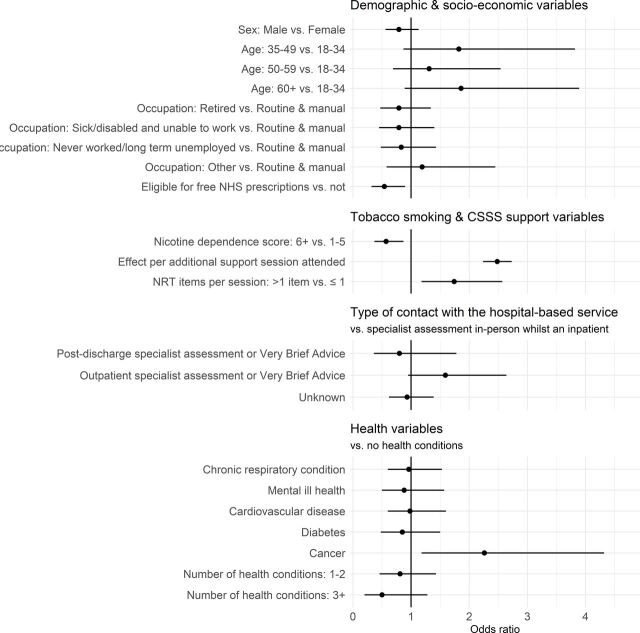
Adjusted ORs (points) with 95% CIs (horizontal lines) showing the relative effects of the explanatory variables from model 5. An effect is statistically significant at the 95% level if the lines showing the CIs do not overlap with 1, where 1 is no effect. CSSS, community stop smoking services; NHS, National Health Service; NRT, nicotine replacement therapy.

Overall, model 5 with the most complex structure also had the best data fit (figure 1; see online supplemental tables S7–S10 for full analysis results).

In model 5, there was no significant difference in 4-week quit success by sex, age or occupation. However, the odds of success by someone eligible (vs not eligible) for free NHS prescriptions were 46% lower (OR 0.54, 95% CI 0.32 to 0.90). Similarly, the odds of success for someone with high (vs low/medium) nicotine dependence were 43% lower (OR 0.57, 95% CI 0.37 to 0.87). Perhaps unsurprisingly, there was strong evidence for higher quitting success among people who attended more CSSS support sessions and who received NRT at a greater intensity of supply. On average, individuals in our analysis sample attended 6 support sessions (range: 1–26). While most people tended to have ≤1 NRT items given per session, 31.0% were on average given more than one type of NRT per session, indicating more intensive pharmacotherapy.

There were no significant effects on quitting success of the type of contact with the hospital service, although there was a trend for outpatients (vs inpatients) to have higher quitting success (OR 1.59, 95% CI 0.95 to 2.64). In our analysis sample, 71.9% of people reported having at least one health condition, with 25.4% of people having three or more conditions. The five most common conditions were chronic respiratory (37.6%), mental health (26.3%), cardiovascular disease (25.2%), diabetes (12.4%) and cancer (9.3%). Compared with having no health conditions, a strong finding of the analysis was that having cancer significantly increased quitting success (OR 2.26, 95% CI 1.18 to 4.32). None of the associations between quitting success and people reporting having any one of the other health conditions reached statistical significance. There was, however, a trend towards people with more comorbid health conditions having lower quitting success, but not with statistical significance (1–2 conditions vs no conditions: OR 0.81, 95% CI 0.46 to 1.43; 3+ conditions vs no conditions: OR 0.50, 95% CI 0.20 to 1.28).

## Discussion

This study investigated the patient flows from an acute hospital inpatient tobacco dependence treatment service to a CSSS, and the subsequent success in quitting smoking. It corresponds to an early phase in the implementation of the hospital service, during and after which there was substantial improvement activity on identification of patients who smoke, the support given and the transfer of care to CSSS on discharge. Of the hospital-referred people who made a CSSS-supported quit attempt, 61% achieved a 4-week quit, slightly below the local average CSSS-supported quit rate. Extrapolating this quit rate to 6 months, based on people who used NRT in clinical trials,^35^ gives a quit rate of 33%, which is similar to the Canadian Ottawa service.^13 14^ However, when patient dropout throughout the entire hospital and CSSS care pathway is factored in, this quit rate falls substantially. The CSSS-supported quit rate of inpatients identified as smoking was 3.3%, rising to 7.7% of inpatients who had a specialist assessment in hospital and 25.3% of inpatients referred to the CSSS. Of the patients who received CSSS support postdischarge, our analysis highlights that those in more vulnerable health and socioeconomic situations, and with higher nicotine dependence, had lower quitting success. However, unlike for other types of health condition, patients who reported having cancer (vs no health conditions) on registering with the CSSS had significantly higher quitting success.

### Implications for service improvement

#### Who might require additional support to quit?

While research has identified factors influencing quitting among people who smoke in general^36^ and those receiving CSSS support,^37 38^ there is limited evidence on how people’s health and hospital treatment are associated with quitting outcomes.^28^ People with poorer health status could have lower capability to quit, although the hospital contact might also be a catalyst that supports quitting.^39^ Previous studies have suggested lower quitting success in patients with higher cardiovascular risk,^40^ more comorbidities^40^ and certain mental health histories.^41–43^ However, an evaluation of tobacco dependence treatment services in two London hospitals found that receiving treatment for a smoking-related disease increased quitting success up to 6 months after discharge.^14^ This suggests that having a smoking-related disease, or receiving a new diagnosis as in cancer screening,^44^ could increase motivation to quit. However, having a smoking-related health condition does not always translate into higher smoking abstinence rates, potentially due to low self-efficacy, not receiving intensive cessation treatments and greater nicotine dependence.^9^ Our findings showed that people who were eligible for free NHS prescriptions—indicating health and/or socio-economic vulnerability—had lower quitting success, matching previous findings from English CSSS.^38^ In addition, while ethnicity in our analysis sample was primarily ‘white’, the evaluation of the tobacco dependence treatment service in the two London hospitals found that people with ‘mixed/Asian/other’ ethnicity had lower quitting success.^14^

#### The importance of flexible and personalised smoking cessation support

As with other acute hospitals,^14 45 46^ the QUIT service experienced challenges with high levels of patient loss to follow-up after leaving hospital.^23^ Improvement initiatives have an important role in helping more patients treated for tobacco dependence in hospital to subsequently receive support in the community after discharge. The conversations in hospital that prompt someone to think about their smoking and increase their motivation to quit are crucial in this respect.^39^ More challenging is understanding the barriers to continuing a quit attempt postdischarge, especially for people who might be acutely unwell. For example, there can be several barriers to cancer patients receiving the right kind of support to quit smoking, even though they are potentially highly motivated.^47^

For services in England, it is recommended that hospital tobacco teams make personalised plans for patients’ ongoing support to stop smoking before discharge, with follow-up calls at 7–14 days and 28 days after discharge.^10^ The discharge plan and follow-up calls provide an opportunity to highlight the flexibility and choice in support options, which could help facilitate engagement.^18^ The CSSS might also benefit from the hospital sharing key patient information, for example, health status and how smoking cessation fits into their care plan; this sharing is already done as part of the QUIT service. The information shared could help the CSSS to personalise their initial approach to patients referred from hospital. Such initiatives to improve the personalisation of care could also help to improve quitting success for other groups supported by hospital tobacco teams, such as children, their parents/carers and NHS staff who smoke.

### Strengths and limitations of the study

The study’s strengths include the systematic approach to analysis development and conduct. Statistical analysis plan development was supported by a rapid systematic literature review to identify factors associated with quitting outcomes.^28^ Further details were then developed through discussions with the QUIT service team,^23^ which addressed constraints on data collection and sharing, data completeness and understanding the collected data fields. The QUIT service and CSSS teams both aided with interpreting the study findings, providing service context.

There were four main study limitations, primarily concerning the data used. First, we could not investigate quitting success beyond 4 weeks, although being smoke-free at 4 weeks is predictive of longer-term success.^35^ Second, sample size was limited by the study only being able to use data from one of two CSSSs receiving patients from the hospitals implementing the QUIT service (due to logistical/contractual reasons). This meant that CSSS referrals from The Rotherham NHS Foundation Trust could not be included in this study. Third, due to CSSS data sharing restrictions, it was not possible to receive individual-level data for people who had not been referred from hospital, which could have allowed for detailed comparisons. Instead, we used summary statistics from the local and national CSSS reporting data as the comparator, although the national data have known limitations.^48^ Fourth, although we intended to, it was not possible for us to link a sufficiently large sample of individuals between the hospital and CSSS records. This meant that it was not possible for our analysis to use information on smoking status and treatment throughout a patient’s stay in hospital, as recommended in the standard evaluation framework for these services (see section 7 of the online supplemental information for further discussion).^49^

## Conclusions

In conclusion, hospitals identifying patients who smoke and then giving treatment for tobacco dependence is likely to increase the number of people quitting smoking. However, the high drop-out rate between hospital referral to postdischarge support to stop smoking in the community and the subsequent uptake of that support is a clear focus for improvement initiatives. Collaboration between hospital and CSSS is key to this improvement, by jointly focusing on optimising the transfer of care and tailoring support to individual needs.

## Data Availability

Data are available on reasonable request. The data underlying this article cannot be shared publicly to protect the privacy of participants. An anonymised version of the data, restricted to include only the variables used for analysis, will be shared on reasonable request to the corresponding author.
