# Supplementary material for: Smoking cessation after referral from hospital to community stop smoking services: an observational study
Source: BMJ Public Health. 2025 Nov 6;3(2):e001659. doi: 10.1136/bmjph-2024-001659 (PMC12658498; doi:10.1136/bmjph-2024-001659)
Supplement: Supplementary data [file bmjph-3-2-s001.pdf]

Supplementary Information for "Smoking Cessation after Referral from Hospital to Community Stop Smoking Services: An Observational Study"

Table of Contents

1 The QUIT hospital-based tobacco dependence treatment programme 2
2 Summary statistics from the NHS Yorkshire Smokefree Community Stop Smoking Service 4
3 National CSSS reporting data 5
4 Further details of explanatory variables for regression analysis 7
5 Descriptive statistics of the analysis sample 10
6 Results of statistical analysis 11
7 Extended implications 15
8 References 17

Cherodian R, *et al. BMJPH* 2025; 3:e001659. doi: 10.1136/bmjph-2024-001659

1 The QUIT hospital-based tobacco dependence treatment programme

The QUIT programme (https://sybics-quit.co.uk) is a major investment across the South Yorkshire Integrated Care Board (ICB), aiming to systematically treat tobacco dependence within secondary care. In 2021, an estimated 16.1% of adults over age 18 in the ICB area were smokers, compared to 13% in England as a whole [1].

QUIT is a complex programme being delivered in partnership with eight NHS hospital Trusts, five community stop smoking services/integrated wellbeing services and community pharmacies. The service spans five local authority areas.

QUIT has four main strands (for a detailed service specification see https://sybics-quit.co.uk/healthcare-professionals/quit-treatment-pathways)

- QUIT for Patients: treatment of tobacco dependence by clinical teams and specialist tobacco treatment advisers.
- QUIT for Parents: clinical teams will provide Very Brief Advice and refer the parents/carers of children who are receiving hospital care to the tobacco treatment advisers.
- QUIT for Staff: on site specialist support from tobacco treatment advisers and free medications for NHS Trust staff who would like to quit.
- QUIT for a Smoke Free Hospital: promoting a smoke-free environment for staff, patients, and visitors.

The QUIT for Patients strand is composed of:

- Acute trust inpatients
- Mental health trust inpatients
- Children’s inpatients
- Secondary care community mental health
- Acute trust: A&E, outpatients and day cases

The scope of this study is limited to inpatients and outpatients from acute hospital Trusts who were referred to one of the community stop services involved.

Cherodian R, *et al. BMJPH* 2025; 3:e001659. doi: 10.1136/bmjph-2024-001659

Trusts implementing each patient pathway.

<table>
<tr><th>Patient pathway</th><th>Trust</th></tr>
<tr><td>Acute Trust: Inpatients, A&E, Outpatients and Day Cases</td><td>• Doncaster and Bassetlaw Hospitals NHS Foundation Trust (DBTH)
• Barnsley Hospitals NHS Foundation Trust (BHNFT)
• The Rotherham Hospital NHS Foundation Trust (TRFT)
• Sheffield Teaching Hospitals NHS Foundation Trust (STH)</td></tr>
<tr><td>Mental Health Trust Inpatients</td><td rowspan="2">• Rotherham, Doncaster, and South Humber NHS Foundation Trust (RDASH) (Rotherham and Doncaster psychiatric sites and services only)
• Sheffield Health and Social Care NHS Foundation Trust (SHSC)
• Southwest Yorkshire Partnership NHS Foundation Trust (Barnsley psychiatric sites and services only) (SWYFT)</td></tr>
<tr><td>Secondary Care Community Mental Health</td></tr>
<tr><td>Children and parents/carers</td><td>• Sheffield Children’s Hospital (SCH)

And children admitted to 4 Acute Trusts:
• Doncaster and Bassetlaw Hospitals NHS Foundation Trust
• Barnsley Hospitals NHS Foundation Trust
• The Rotherham Hospital NHS Foundation Trust
• Sheffield Teaching Hospitals NHS Foundation Trust</td></tr>
</table>

The letters in QUIT stand for the key steps taken by ward staff to start someone’s tobacco dependence treatment when they have been admitted to hospital.

Q	Ask the **Q**uestion	Ask all hospital patients is they have smoked in the past month.
U	**U**nderstand their addiction	Ask all hospital patients to exhale into a Carbon Monoxide breathalyser.<br><br>Ask patients who smoke about how much they smoke to help work out how much Nicotine Replacement Therapy (NRT) to give to those aged 12 and over.
I	**I**nform patients	About smoke-free sites & that specialist support is available while they are in hospital.
T	Initiate **T**reatment	Offer all smokers NRT on admission & notify the tobacco treatment advisors of all smokers so they can provide specialist support and treatment as soon as possible.

Cherodian R, *et al. BMJPH* 2025; 3:e001659. doi: 10.1136/bmjph-2024-001659

2 Summary statistics from the NHS Yorkshire Smokefree Community Stop Smoking Service

The NHS Yorkshire Smokefree Community Stop Smoking Service (CSSS) provided summary statistics on the numbers of people referred to and engaging with their service, in total and from the QUIT service from July 2021 to March 2023.

Table S1. Monthly statistics showing total CSSS activity and the percentage of that activity due to referrals from the QUIT service.

	Total CSSS clients			Clients referred from the QUIT service			Percentage of clients referred from the QUIT service		
**Month**	**Referrals**	**Set a quit date**	**Achieved a 4-week quit**	**Referrals**	**Set a quit date**	**Achieved a 4-week quit**	**Referrals**	**Set a quit date**	**Achieved a 4-week quit**
**Jul-21**	667	511	327	56	21	12	8%	4%	4%
**Aug-21**	748	460	290	70	29	19	9%	6%	7%
**Sep-21**	870	427	288	59	13	9	7%	3%	3%
**Oct-21**	841	497	331	114	35	29	14%	7%	9%
**Nov-21**	846	522	347	100	45	30	12%	9%	9%
**Dec-21**	472	329	219	107	36	27	23%	11%	12%
**Jan-22**	975	541	375	131	48	24	13%	9%	6%
**Feb-22**	728	465	331	137	63	38	19%	14%	11%
**Mar-22**	807	420	310	154	73	41	19%	17%	13%
**Apr-22**	670	418	303	181	71	47	27%	17%	16%
**May-22**	778	464	292	208	92	58	27%	20%	20%
**Jun-22**	718	398	246	180	65	26	25%	16%	11%
**Jul-22**	722	411	275	193	72	36	27%	18%	13%
**Aug-22**	769	412	285	194	82	43	25%	20%	15%
**Sep-22**	718	369	272	178	70	43	25%	19%	16%
**Oct-22**	727	440	300	166	69	47	23%	16%	16%
**Nov-22**	693	427	274	200	88	52	29%	21%	19%
**Dec-22**	488	295	198	170	78	53	35%	26%	27%
**Jan-23**	961	508	341	228	96	60	24%	19%	18%
**Feb-23**	799	437	297	198	87	58	25%	20%	20%
**Mar-23**	896	550	352	199	100	63	22%	18%	18%
**Total**	15,893	9,301	6,253	3,223	1,333	815	20%	14%	13%

Cherodian R, *et al. BMJPH* 2025; 3:e001659. doi: 10.1136/bmjph-2024-001659

3 National CSSS reporting data

Collected by NHS England (previously NHS Digital) from local government commissioned services every 3-months, the data includes the number of quit attempts made and that led to 4-week quits (self-reported and CO verified), individual characteristics (e.g., smoking-related details), and the type of support received [2]. Local profiles were obtained by selecting only the localities covered by the CSSS in this study: Barnsley (identifier E08000016), Doncaster (E08000017), and Sheffield (E08000019), which contained a mix of data from the NHS Yorkshire Smokefree CSSS and other local services, e.g., specialist maternity stop smoking services. However, since 2014, activity reporting by CSSSs to NHS England has been voluntary with no data collection standards; for 2022 to 2023, 15 of 151 local areas did not submit data [3].

Table S2. Quit attempts. Age, sex and socio-economic profile of the number of quit dates set. Comparison between the analysis sample of quit attempts (1st July 2021 to 31st March 2023) and national and local samples (1st April 2022 to 31st March 2023).

	**Analysis sample**		**National**		**Local [3]**	
	**Number of observations [2]**	**Percentage of total sample**	**Number of observations**	**Percentage of total sample**	**Number of observations**	**Percentage of total sample**
Total	1,326		176,566		5,083	
Male						
Under 18 [1]	-	-	484	0.3%	15	0.3%
18–34	62	4.7%	11,640	6.6%	346	6.8%
35–44	102	7.7%	15,120	8.6%	391	7.7%
45–59	273	20.6%	28,761	16.3%	832	16.4%
60 and over	254	19.2%	24,045	13.6%	709	13.9%
Female						
Under 18	-	-	571	0.3%	21	0.4%
18–34	50	3.8%	23,445	13.3%	585	11.5%
35–44	88	6.6%	17,626	10.0%	452	8.9%
45–59	231	17.4%	29,705	16.8%	868	17.1%
60 and over	266	20.1%	25,169	14.3%	864	17.0%
Occupation						
Routine and manual occupations	411	31.0%	42,394	24.0%	1,644	32.3%
Retired	321	24.2%	25,642	14.5%	908	17.9%
Sick/disabled and unable to work	296	22.3%	22,233	12.6%	839	16.5%
Never worked or unemployed for over 1 year	196	14.8%	26,376	14.9%	851	16.7%
Other	102	7.7%	59,921	33.9%	841	16.5%
Eligible for free NHS prescriptions						
Yes	1,076	81.1%	109,015	61.7%	3,034	59.7%

[1] The analysis sample was restricted to individuals 18 years and over, excluding 2 individuals.
[2] The number of observations does not necessarily equal the number of individuals because individuals could make more than one quit attempt.
[3] Local profiles were obtained by selecting only the localities covered by the CSSS in this study: Barnsley (identifier E08000016), Doncaster (E08000017) and Sheffield (E08000019).

Cherodian R, *et al. BMJPH* 2025; 3:e001659. doi: 10.1136/bmjph-2024-001659

Table S3. Quit success. Age, sex and socio-economic profile of the number of 4-week quits. Comparison between the analysis sample (1st July 2021 to 31st March 2023) and national and local samples (1st April 2022 to 31st March 2023).

	**Analysis sample**		**National**		**Local** [3]	
	**Number of 4-week quits** [1]	**Percentage of quit attempts**	**Number of 4-week quits**	**Percentage of quit attempts**	**Number of 4-week quits**	**Percentage of quit attempts**
Total	813	61.3%	109,356	61.9%	3,540	69.6%
Male						
Under 18	-	-	269	55.6%	11	73.3%
18–34	29	46.8%	6,943	59.6%	222	64.2%
35–44	57	55.9%	9,379	62.0%	254	65.0%
45–59	167	61.2%	18,436	64.1%	566	68.0%
60 and over	177	69.7%	16,176	67.3%	529	74.6%
Female						
Under 18	-	-	353	61.8%	23	109.5% [3]
18–34	24	48.0%	13,768	58.7%	509	87.0%
35–44	53	60.2%	10,418	59.1%	309	68.4%
45–59	137	59.3%	17,925	60.3%	532	61.3%
60 and over	169	63.5%	15,689	62.3%	585	67.7%
Occupation [2]						
Routine and manual occupations	254	61.8%	24,114	56.9%	1,084	65.9%
Retired	213	66.4%	14,701	57.3%	628	69.2%
Sick/disabled and unable to work	171	57.8%	11,753	52.9%	514	61.3%
Never worked or unemployed for over 1 year	104	53.1%	12,716	48.2%	497	58.4%
Other	71	69.6%	32,116	53.6%	539	64.1%
Eligible for free NHS prescriptions						
Yes	649	60.3%	58,906	54.0%	1,889	62.3%

[1] The sum of self-reported and carbon monoxide verified 4-week quits.
[2] Only 87.2% (95,400 / 109,356) of 4-week quits in the national data had data on occupation; 4-week quits without occupation are not reported.
[3] A more than 100% quit success is likely to be due to data recording error, which is made more obvious due to the small number of quit attempts.

Cherodian R, *et al. BMJPH* 2025; 3:e001659. doi: 10.1136/bmjph-2024-001659

4 Further details of explanatory variables for regression analysis

Table S4. Socio-economic classifications used in the stop smoking service data. Categories were merged due to limited sample size.

Variable definitions in the service monitoring data[1]	Analysis category
Routine and manual (self-employed should not be included in this category). Examples include: electrician, fitter, gardener, inspector, plumber, printer, train driver, tool maker, bar staff, caretaker, catering assistant, cleaner, farm worker, HGV driver, labourer, machine operative, messenger, packer, porter, postal worker, receptionist, sales assistant, security guard, sewing machinist, van driver, waiter/waitress.	Routine and manual occupations
Retired	Retired
Sick or disabled and unable to work	Sick or disabled and unable to work
Never worked or unemployed for over one year. If unemployed for less than one year, last known occupation should be used for classification.	Never worked or unemployed for over 1 year
Full-time Student	Other
Home carer i.e. looking after children, family or home	Other
Managerial/ professional. Examples include: accountant, artist, civil/mechanical engineer, medical practitioner, musician, nurse, police officer (sergeant or above), physiotherapist, scientist, social worker, software engineer, solicitor, teacher, welfare officer. Those usually responsible for planning, organising and co-ordinating work for finance	Other
Intermediate. Examples include: call centre agent, clerical worker, nursery auxiliary, office clerk, secretary	Other
Prisoner	Other
Other (not listed)	Other

[1] Available from: https://www.datadictionary.nhs.uk/data_elements/socio-economic_classification_code__stop_smoking_.html

Table S5. Names of the 16 medical conditions available in the data and their grouping to form the 5 categories of disease that were used for analysis.

**Medical condition**	**Category**
Angina	Cardiovascular disease
Heart attack	Cardiovascular disease
Heart disease / stroke	Cardiovascular disease
Diabetes	Diabetes
Depression	Mental ill health
Anxiety	Mental ill health
Severe mental illness	Mental ill health
Other long-term mental health condition	Mental ill health
Cancer	Cancer
Asthma	Chronic respiratory conditions
Chronic obstructive pulmonary disease	Chronic respiratory conditions
Epilepsy	Not investigated individually due to insufficient data but used in the description of multi-morbidity.
Renal disease	Not investigated individually due to insufficient data but used in the description of multi-morbidity.
Thyroid - overactive	Not investigated individually due to insufficient data but used in the description of multi-morbidity.
Post & pre-operative	Not investigated individually due to insufficient data but used in the description of multi-morbidity.
Any other long-term condition	Not investigated individually due to insufficient data but used in the description of multi-morbidity.

Table S6. Details for why some variables that were intended to be included in the analysis were not included.

**Variable intended for inclusion** [4]	**Reason not included**
Index of Multiple Deprivation (IMD) (a small-area geographic indicator of socio-economic conditions) [5]	Postcode information only provided to the level of sector of residence. Full postcode needed to link to IMD.
Ethnicity	Available as a 17-level classification.<br><br>Not usable because majority white or unknown.<br><br>Sample: 85% (1,425 / 1,677) white, 11% (186 / 1,677) missing.
Maternity status	Available as pregnant yes/no. Not usable because majority no.
Lives with other smokers	Available as yes/no. Not usable because 41% missing.
Number of previous attempts to stop smoking	Not available
Type of inpatient admission (elective/emergency)	CSSS data could not be linked to inpatient hospital records.
Speciality of the medical consultant caring for the patient	CSSS data could not be linked to inpatient hospital records.
Primary diagnosis (the diagnosis assigned on admission to hospital)	CSSS data could not be linked to inpatient hospital records.
Whether on medication whose metabolism was affected by smoking	Available as yes/no. Not usable because 94% missing.
Whether attained a continuous four-week period of abstinence while hospitalised	Available as yes/no. Not usable because 97% missing.

Cherodian R, *et al. BMJPH* 2025; 3:e001659. doi: 10.1136/bmjph-2024-001659

5 Descriptive statistics of the analysis sample

Figure S1 shows monthly counts of all hospital-referred individuals registered with the CSSS, of individuals in the analysis sample, and of individuals who achieved a 4-week quit.

Figure S1: Description of the CSSS data sample—Monthly counts of registered, in-analysis, and 4 week quits.

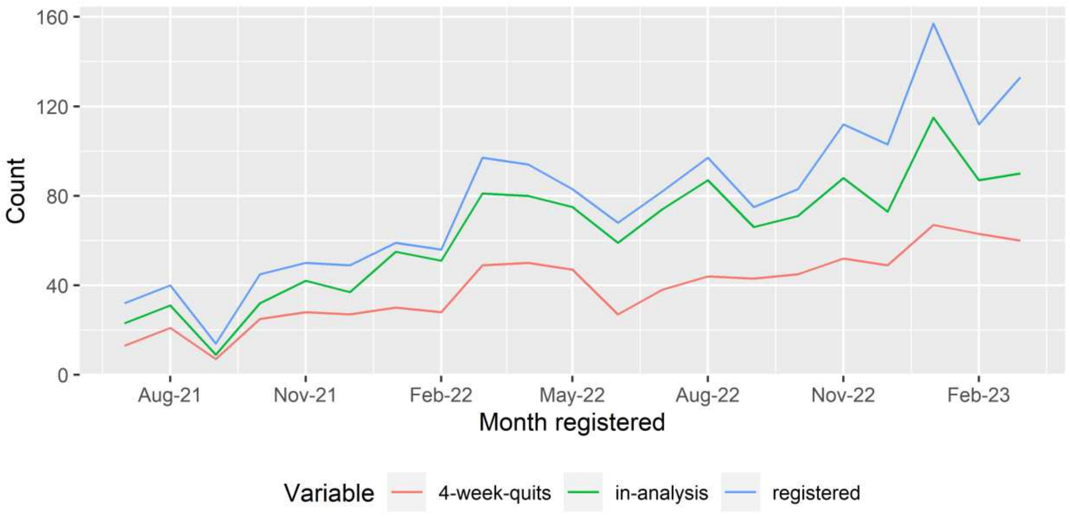

Cherodian R, *et al. BMJPH* 2025; 3:e001659. doi: 10.1136/bmjph-2024-001659

6 Results of statistical analysis

The analysis was based on fitting five alternative model structures which sequentially added explanatory variables:

- (Model-1) individual demographic and socio-economic characteristics;
- (Model-2) Model-1 with the Fagerström score for nicotine dependence;
- (Model-3) Model-2 with CSSS support (support sessions and pharmacotherapy);
- (Model-4) Model-3 with hospital contact type (inpatient vs. post-discharge vs. outpatient);
- (Model 5) Model-4 with health co-morbidities.

Table S7 compares the fits of these models. The main results text presents findings from the best fitting model – Model 5. Table S8 presents the coefficients from each model. Table S9 presents sensitivity analyses of the estimates in Model 5. Table S10 shows the percentage quitting success according to the health factors investigated to support interpretation of the findings.

Table S7. The five alternative model structures investigated and statistics showing comparative model fit.

**Model:**	**1**	**2**	**3**	**4**	**5**
**Model structure**					
Demographic and socio-economic variables	Included	Included	Included	Included	Included
Strength of nicotine dependence		Included	Included	Included	Included
CSSS support			Included	Included	Included
Type of contact with the hospital-based service				Included	Included
Health variables					Included
**Akaike's Information Criterion** [1]	1759.2	1761.0	922.1	924.0	921.7
**P-values from Likelihood Ratio Tests of the difference in residual deviance between models**					
vs. Model 1		0.365	***0.000***	***0.000***	***0.000***
vs. Model 2			***0.000***	***0.000***	***0.000***
vs. Model 3				0.762	***0.005***
vs. Model 4					***0.023***

[1] Smaller values of Akaike's Information Criterion indicate a better model fit to the data.

Table S8. Coefficient estimates (odds ratios) from the five model structures considered. Numbers in parenthesis are 95% confidence intervals.

Model:	1	2	3	4	5
Constant	1.10 (0.69,1.74)	1.09 (0.68,1.75)	4.76 (2.25,10.04)	4.55 (2.10,9.82)	6.49 (2.84,14.85)
**Demographic and socio-economic variables**					
Male (vs. Female)	1.10 (0.88,1.38)	1.10 (0.88,1.38)	0.87 (0.62,1.22)	0.87 (0.62,1.22)	0.79 (0.56,1.13)
Age group (vs. 18–34)					
35–49	1.51 (0.94,2.44)	1.51 (0.94,2.44)	***2.11 (1.02,4.36)***	***2.10 (1.01,4.36)***	1.82 (0.87,3.82)
50–59	***1.74 (1.14,2.65)***	***1.73 (1.14,2.65)***	1.41 (0.74,2.68)	1.36 (0.71,2.59)	1.31 (0.68,2.54)
60+	***2.42 (1.51,3.88)***	***2.42 (1.51,3.88)***	2.04 (1.00,4.17)	2.01 (0.99,4.11)	1.86 (0.89,3.89)
Occupation (vs. Routine and manual)					
Retired	1.00 (0.68,1.46)	0.99 (0.67,1.47)	0.77 (0.44,1.34)	0.76 (0.43,1.32)	0.79 (0.45,1.40)
Sick/disabled and unable to work	0.91 (0.65,1.26)	0.90 (0.65,1.26)	0.68 (0.41,1.13)	0.69 (0.41,1.16)	0.79 (0.47,1.34)
Never worked/long term unemployed	0.78 (0.54,1.13)	0.78 (0.54,1.13)	0.77 (0.45,1.30)	0.74 (0.43,1.26)	0.83 (0.48,1.43)
Other	1.47 (0.92,2.38)	1.46 (0.91,2.36)	1.14 (0.56,2.30)	1.18 (0.58,2.39)	1.19 (0.58,2.45)
Eligible for free NHS prescriptions (vs. not)	0.73 (0.52,1.01)	0.73 (0.52,1.02)	***0.54 (0.33,0.88)***	***0.54 (0.33,0.89)***	***0.54 (0.32,0.90)***
**Strength of nicotine dependence (Fagerström score)** [1]					
6+ (vs. 1–5)		1.02 (0.80,1.31)	***0.50 (0.34,0.74)***	***0.52 (0.34,0.78)***	***0.57 (0.37,0.87)***
**CSSS support**					
Effect per additional support session attended			***2.39 (2.17,2.62)***	***2.40 (2.18,2.64)***	***2.48 (2.24,2.73)***
NRT intensity index (items per session)					
>1 item (vs. ≤1 item)			***1.73 (1.17,2.54)***	***1.71 (1.16,2.52)***	***1.74 (1.18,2.57)***
**Type of contact with the hospital-based service** (vs. Specialist assessment in-person whilst an inpatient)					
Post-discharge specialist assessment or Very Brief Advice				0.91 (0.41,2.00)	0.80 (0.36,1.78)
Outpatient specialist assessment or Very Brief Advice				1.60 (0.97,2.64)	1.59 (0.95,2.64)
Unknown				0.97 (0.66,1.44)	0.93 (0.62,1.39)
**Health variables**					
Specific health conditions (vs. no health conditions)					
Chronic respiratory condition					0.96 (0.60,1.53)
Mental ill health					0.88 (0.50,1.57)
Cardiovascular disease					0.98 (0.60,1.60)
Diabetes					0.85 (0.48,1.50)
Cancer					***2.26 (1.18,4.32)***
Number of health conditions (vs. no health conditions)					
1–2					0.81 (0.46,1.43)
3+					0.50 (0.20,1.28)

[1] Missing values imputed; estimates generated from 19 imputed data sets, each with N = 1,326.

Cherodian R, *et al. BMJPH* 2025; 3:e001659. doi: 10.1136/bmjph-2024-001659

Table S9. Sensitivity analysis of the parameter estimates in the base case version of Model 5 (1), to the effects of only including people with complete information on the Fagerström score of nicotine dependence (2), and omitting people lost to follow-up (3).

	Base case (1)	Complete Fagerström score (2)	Complete follow-up (3)
Constant	6.49 (2.84,14.85)	0.01 (0.00,0.03)	0.03 (0.01,0.08)
**Demographic and socio-economic variables**			
Male (vs. Female)	0.79 (0.56,1.13)	0.77 (0.51,1.15)	0.85 (0.59,1.23)
Age group, reference 18–34			
35–49	1.82 (0.87,3.82)	1.48 (0.64,3.47)	1.80 (0.81,4.01)
50–59	1.31 (0.68,2.54)	0.93 (0.44,1.99)	1.24 (0.61,2.52)
60+	1.86 (0.89,3.89)	1.17 (0.50,2.76)	1.56 (0.72,3.41)
Occupation (vs. Routine and manual)			
Retired	0.79 (0.45,1.40)	0.78 (0.41,1.48)	0.81 (0.45,1.46)
Sick/disabled and unable to work	0.79 (0.47,1.34)	0.91 (0.49,1.67)	0.85 (0.49,1.48)
Never worked/long term unemployed	0.83 (0.48,1.43)	0.91 (0.49,1.68)	0.74 (0.42,1.31)
Other	1.19 (0.58,2.45)	1.08 (0.47,2.53)	1.38 (0.63,2.99)
Eligible for free NHS prescriptions (vs. not)	***0.54 (0.32,0.90)***	0.58 (0.32,1.05)	***0.57 (0.33,0.97)***
**Strength of nicotine dependence (Fagerström score)**			
6+ (vs. 1–5)	***0.57 (0.37,0.87)***	***0.58 (0.38,0.88)***	***0.51 (0.33,0.78)***
**CSSS support**			
Effect per additional support session attended	***2.48 (2.24,2.73)***	***2.82 (2.50,3.22)***	***2.38 (2.15,2.64)***
NRT items per session			
>1 item (vs. ≤1 item)	***1.74 (1.18,2.57)***	***1.87 (1.19,2.97)***	***1.98 (1.30,3.02)***
**Type of contact with the hospital-based service** (vs. Specialist assessment in-person whilst an inpatient)			
QUIT type, reference inpatient; full assessment			
Post-discharge specialist assessment or Very Brief Advice	0.80 (0.36,1.78)	0.70 (0.28,1.70)	0.74 (0.32,1.69)
Outpatient specialist assessment or Very Brief Advice	1.59 (0.95,2.64)	1.46 (0.81,2.64)	1.38 (0.82,2.31)
Unknown	0.93 (0.62,1.39)	0.79 (0.49,1.25)	1.00 (0.65,1.53)
**Health variables**			
Specific health conditions (vs. no health conditions)			
Chronic respiratory condition	0.96 (0.60,1.53)	0.90 (0.54,1.51)	1.01 (0.62,1.63)
Mental ill health	0.88 (0.50,1.57)	0.76 (0.39,1.47)	0.94 (0.51,1.71)
Cardiovascular disease	0.98 (0.60,1.60)	0.91 (0.52,1.58)	0.90 (0.54,1.49)
Diabetes	0.85 (0.48,1.50)	0.97 (0.51,1.83)	0.93 (0.50,1.70)
Cancer	***2.26 (1.18,4.32)***	***2.12 (1.01,4.52)***	1.94 (0.99,3.80)
Number of health conditions (vs. no health conditions)			
1–2	0.81 (0.46,1.43)	0.91 (0.46,1.78)	0.78 (0.42,1.42)
3+	0.50 (0.20,1.28)	0.50 (0.17,1.45)	0.46 (0.17,1.23)
Lost to follow up	not quit	not quit	omitted
Fagerström dummy imputed	yes	no	yes
N	1,326	1,074	1,195

Estimates are odds ratios. Figures in parenthesis are 95% confidence intervals.

Cherodian R, *et al. BMJPH* 2025; 3:e001659. doi: 10.1136/bmjph-2024-001659

Table S10. The percentages of people who made a quit attempt with CSSS, in the analysis data sample, who achieved a 4-week quit according to nature of contact with the hospital-based (QUIT) service and health status.

**Type of contact with the hospital-based service**	**% of 4 week quits** (assuming lost to follow-up is not quit)	**% of 4 week quits** (excluding lost to follow-up from the denominator)
Specialist assessment in-person whilst an inpatient	57.0%	65.0%
Specialist assessment / Very Brief Advice post-discharge	50.0%	57.9%
Specialist assessment / Very Brief Advice outpatient	61.4%	63.9%
Unknown	66.8%	73.8%
**Health variables**		
Specific health conditions		
Chronic respiratory condition	60.2%	65.9%
Mental ill health	62.6%	65.9%
Cardiovascular disease	66.7%	70.9%
Diabetes	60.0%	68.8%
Cancer	69.6%	73.1%
Number of health conditions		
0	55.6%	64.8%
1–2	64.6%	70.3%
3+	60.7%	66.6%

7 Extended implications

Implications for service monitoring and evaluation

Quitting outcomes

The findings of this study offer a snapshot of the quits obtained via CSSS in an early period of QUIT service implementation, during which the hospital and CSSS teams were actively implementing improvements, particularly in facilitating the effective transfer of patients to CSSS. Further data collection is important to evaluate the service's efficacy once it is fully embedded in the hospital culture and more issues are addressed. A larger dataset would also provide greater statistical power to detect variations in quitting outcomes based on patient and service characteristics.

Monitoring quitting outcomes presents challenges due to differing levels of engagement with both hospital-based services and CSSS. Of the inpatients who received a specialist assessment for tobacco dependence in hospital during the study period, only a third were recorded in the Key Performance Indicator data for the QUIT service to have been referred on to CSSS, which could have been due to them declining or referrals not being recorded. Quitting outcomes in the remainder, i.e. people who were not referred to a CSSS or who were referred but did not engage with the CSSS, have to be ascertained by the hospital tobacco teams in their 4-week post-discharge follow-up appointments, with quitting outcome data from these follow-ups not available for analysis. In addition, quitting outcomes for outpatients and those receiving only Very Brief Advice in hospital could remain unknown unless they go on to take up support from CSSS, as some did in our data sample. The overall quits achieved by the QUIT service will depend on the quitting outcomes of patients who receive all types of post-discharge support, including from CSSS, community pharmacies, and self-quitting.[6] Furthermore, the family and friends of individuals who received support from the QUIT service might have been motivated to quit, but no related data is available. However, as more people arrive at CSSS from hospital, the more representative CSSS outcomes will become of the hospital-based service as a whole. To prevent people from dropping out of smoking cessation support after leaving the hospital and to keep them motivated throughout the process, the QUIT service is exploring new strategies. One approach is to have hospital-based services book CSSS appointments for patients immediately, rather than relying on the CSSS to contact patients after discharge. Additionally, having in-reach CSSS advisors in the hospital to facilitate transfers is being considered.

Linking patient hospital and CSSS records

Being able to track the experiences of individuals across hospital and CSSS records could help to understand how improvements to the hospital-based service affect the experiences of people attending CSSS. The original evaluation plan was to analyse predictors of quitting outcomes using a dataset that linked individual records from the hospital-based service to CSSS records using each patient's unique NHS number. This linkage would have allowed for additional explanatory variables to be drawn from the hospital records, adding to the information from the hospital records that was already transferred to the CSSS on patient referral. However, the number of individuals who could be linked between the two record systems was too limited to provide a sample size large enough for meaningful analysis: out of 1,641 individuals in the CSSS data, only 786 had a unique identifier that could be used for linkage. Allowing for a three-week window between hospital discharge and CSSS registration, only 332 individuals could then be linked between the two record systems. Therefore, the explanatory variables for this study were limited to CSSS data only;

Supplementary Table S6 gives details for why some intended variables were not included. Improving this data linkage could be beneficial for future service evaluation.

Monitoring community stop smoking service workload

Although there are opportunities to support quit attempts by transferring people from hospitals to CSSSs, CSSSs need sufficient staff to deal with the increased number of clients for the service to work appropriately. To estimate demand from our study's findings, we used summary statistics from the CSSS on the number of hospital-referred clients they supported in relation to their total activity. After an initial phase-in, hospital-referred quit attempts accounted for 19% of all quit attempts supported by CSSS, equating to 81 people who attempted to quit who might not have done so without the QUIT service. However, this calculation does not consider the additional work required from CSSS to contact all hospital-referred patients, of whom only 42% (81/191) went on to make a supported attempt to stop smoking.

Cherodian R, *et al. BMJPH* 2025; 3:e001659. doi: 10.1136/bmjph-2024-001659

8 References

1. Office for Health Improvement & Disparities: **Local Tobacco Control Profiles: Smoking prevalence in adults age 18+ in the South Yorkshire Integrated Care Board**. 2023, url: https://fingertips.phe.org.uk/profile/tobacco-control/data#page/1/gid/1938132885/pat/15/ati/221/are/nE54000061/iid/92443/age/168/sex/4/cat/-1/ctp/-1/yrr/1/cid/4/tbm/1.

2. NHS Digital: **Statistics on NHS Stop Smoking Services in England April 2022 to March 2023 - Data tables**. 2023, url: https://digital.nhs.uk/data-and-information/publications/statistical/statistics-on-nhs-stop-smoking-services-in-england/april-2022-to-march-2023-q4/datasets.

3. McNeill A, Brose L, Robson D, Calder R, Simonavicius E, East K, Taylor E, Zuikova E: **Nicotine vaping in England: an evidence update including health risks and perceptions, 2022**. 2022, url: https://assets.publishing.service.gov.uk/media/633469fc8fa8f5066d28e1a2/Nicotine-vaping-in-England-2022-report.pdf.

4. Gillespie D, Baxter S, Franklin M, Hock E, Flight L, Bright S, Chilcott J: **Evaluation of the QUIT hospital-based smoking cessation service: Interim report - covering the setup phase of the evaluation**. 2023, url: https://doi.org/10.15131/shef.data.23622864.

5. Department for Communities and Local Government: **The English Indices of Deprivation 2015**. 2015, url: https://www.gov.uk/government/statistics/english-indices-of-deprivation-2015.

6. National Centre for Smoking Cessation and Training: **NHS Standard Treatment Plan for Inpatient Tobacco Dependence: A guide to support delivery of the Inpatient Tobacco Dependence Treatment Care Bundle**. 2023, url: https://www.ncsct.co.uk/library/view/pdf/NHS-Standard-Treatment-Plan-for-Acute-Inpatient-Tobacco-Dependence.pdf.

Cherodian R, *et al. BMJPH* 2025; 3:e001659. doi: 10.1136/bmjph-2024-001659
